# Overexpression of the cotton trihelix transcription factor *GhGT23* in *Arabidopsis* mediates salt and drought stress tolerance by binding to GT and MYB promoter elements in stress-related genes

**DOI:** 10.3389/fpls.2023.1144650

**Published:** 2023-03-02

**Authors:** Yue Li, Ziyao Hu, Yongmei Dong, Zongming Xie

**Affiliations:** ^1^ College of Life Science, Xinjiang Agricultural University, Urumqi, China; ^2^ Xinjiang Production and Construction Group Key Laboratory of Crop Germplasm Enhancement and Gene Resources Utilization, Xinjiang Academy of Agricultural and Reclamation Science, Shihezi, China

**Keywords:** cotton, abiotic stress, trihelix transcription factor, GhGT23, drought tolerance, salt tolerance, stress-related gene

## Abstract

Cotton (*Gossypium hirsutum* L.) is the world’s most economically valuable textile crop. However, cotton plants are often subjected to numerous abiotic stresses that can dramatically limit yield. Trihelix transcription factors (TTFs) play important roles in abiotic stress responses in many plant species, and efforts to better understand their roles in cotton abiotic stress responses are ongoing. In this study, a member of the cotton TTF family (GhGT23) was functionally characterized. This protein contains a SANT domain and is a member of the SIP subfamily of TTF proteins. *GhGT23* was significantly (p < 0.05) and highly expressed in cotton fiber compared to relatively low expression in other tissues. A significant (p < 0.05) increase in *GhGT23* expression occurred in cotton seedlings within 12 hours of drought, salt, and ABA exposure. The GhGT23 protein localized in the nucleus but exhibited no signs of transactivation activity. *GhGT23* overexpression in *Arabidopsis* conferred enhanced drought and salt stress tolerance. The expression of stress-related genes was higher in transgenic *Arabidopsis* expressing *GhGT23* than in wild-type plants subjected to salt stress. The results of electrophoretic mobility shift assay revealed that GhGT23 could bind to the GT cis-elements GT-1Box (Box II), GT2-Box, GT3-Box, GT-3a (Site1-type), GT-3b, and Box as well as the MYB cis-elements MBS1 and MRE4. Our results demonstrate that *GhGT2*3 positively regulates salt and drought stress responses, possibly by enhancing the expression of stress-related genes.

## Introduction

Plant growth and crop production are constantly challenged by dynamic abiotic and biotic stresses in the environment. Some of the most common abiotic stresses that plants are subjected to are high salt, low temperature, and drought. These abiotic stresses can limit crop productivity and reduce average crop yields by more than 50% (Lesk et al., 2016). Plant cells perceive environmental stress through sensors that trigger signaling pathways upon stress perception. These signaling pathways often include phosphorylation cascades that culminate in the activation of stress-responsive transcription factors (TFs). Upon activation, TFs translocate into the nucleus where they bind to cis-regulatory elements in the promoter regions of several stress-related genes (Zhu, 2016). In this way, TFs play a critical role in translating the initial perception of stress by the plant into physiological changes that result in tolerance to the stress (Basu et al., 2016). Of the more than 64 transcription factor families identified in plants, the NAC, C_2_H_2_ zinc finger, bZIP, and WRKY families are the most studied and most widely involved in abiotic stress responses (Olsen et al., 2005; Pérez-Rodríguez et al., 2010; Llorca et al., 2014). Because of their documented roles in plant stress tolerance, TFs have become key targets for genetic engineering efforts to improve plant stress tolerance.

Trihelix transcription factors (TTFs) are a small family of plant-specific TFs that are present in diverse plant species (Luo et al., 2012). Chrysanthemum, tomato, rice, *Arabidopsis*, poplar, soybean, and maize each have 20, 36, 31, 28, 56, 63, and 59 TTF genes, respectively (Fang et al., 2010; Osorio et al., 2012; Yu et al., 2015; Du et al., 2016; Erum et al., 2016; Song et al., 2016; Wang et al., 2016). Members of the TTF family are also known as GT factors, because they can bind to the GT element found in the promoter of some genes regulated by light (Zhou, 1999). The first GT element was identified in the *rbcS-3A* gene promoter from pea in 1987 (Green et al., 1987). Since then, GT elements have been identified in soybean, maize, spinach, *Arabidopsis*, and rice genes (Dehesh et al., 1990; Lawton et al., 1991; Dehesh et al., 1992; Kuhn et al., 1993; Villain et al., 1994; Eyal et al., 1995; Villain et al., 1996; Ayadi et al., 2004; Park et al., 2004).

The DNA-binding domains of TTFs are rich in proline as well as basic and acidic amino acids. They also contain three tandem helices, namely, the helix-loop-helix-loop-helix (Dehesh et al., 1992; Kuhn et al., 1993; Wang et al., 2014). The internal hydrophobic region of each helix contains regularly spaced repeats typically containing three tryptophan residues separated by a non-tryptophan residue (W-X-W-X-W). The third tryptophan residue is less conserved and is sometimes replaced with a phenylalanine or isoleucine residue (Kaplan-Levy et al., 2012). TTFs contain one or two trihelix DNA-binding domains at the N- or C-terminus that specifically bind to GT-elements (Dehesh et al., 1992; Zhou, 1999). The *Arabidopsis* TTF family is divided into five subfamilies, namely, GT-1, GT-2, GTγ, SH4, and SIP1 (Kaplan-Levy et al., 2012). Most subfamilies contain only one DNA-binding domain, but members of the GT-2 subfamily contain a DNA-binding domain at both the N- and C-terminus. The five subfamilies are further differentiated by the composition of the tryptophan repeats in each helix. Helixes in members of the GT-1 and SH4 subfamily, as well as the C-terminal DNA-binding domain of GT-2 subfamily members, contain a tryptophan residue at the end of the repeat (W-X-W-X-W). On the other hand, members of the GTγ subfamily, and the N-terminal DNA-binding domain of GT-2 subfamily members, contain a phenylalanine residue at the end of the repeat (W-X-W-X-F), whereas members of the SIP1 subfamily contain an isoleucine residue at the terminal position (W-X-W-X-I) (Kaplan-Levy et al., 2012).

Numerous plant TTFs, and the GT elements that they bind, have been reported to regulate responses to light, growth, and a variety of developmental processes such as morphogenesis of perianth organs, formation of trichomes and stomata, seed oil accumulation and abscission, kernel development, and late embryogenesis development (Willmann et al., 2011; Kaplan-Levy et al., 2012; Kaplan-Levy et al., 2014; Wan et al., 2015; Li et al., 2021; Yang et al., 2022). Recent studies on TTF family members in *Arabidopsis*, rice, soybean, wheat, tomato, chrysanthemum, and poplar have revealed that they are widely involved in plant responses to abiotic stress (Lu et al., 2019). Transgenic *Arabidopsis* overexpressing *GT-4* (Wang et al., 2014), *AST1* (Xu et al., 2018), or *AtGTL1* (Yoo et al., 2010) exhibited improved salt and drought stress tolerance, whereas *GmGT2A* and *GmGT2B* expression was induced by high salinity, drought, cold, and abscisic acid (ABA) in soybean, and heterologous overexpression of these two genes in *Arabidopsis* improved salt, cold, and drought stress tolerance (Xie et al., 2009). Furthermore, overexpression of the rice genes *OsGT-1* and *OsGTgamma-2* in rice confered enhanced resistance to salt stress (Fang et al., 2010; Liu et al., 2020). Heterologous expression of wheat *TaGT2L1D* in *Arabidopsis* can suppress the expression of *AtSDD1*, a positive regulator of drought tolerance, by binding directly to the GT3 box in its promoter (Zheng et al., 2016). Overexpression of the sorghum TTF genes *sb06g023980* and *sb06g024110* significantly enhanced tolerance of low temperature, high salt, and drought (Qin et al., 2014), whereas transgenic tomatoes overexpressing *ShCIGT* gained tolerance of low temperature and drought (Yu et al., 2018). Expression of poplar *PtaGTL1* in *Arabidopsis* reduced stomatal conductance and leaf transpiration, which led to enhanced drought tolerance and water use efficiency (Weng et al., 2012). Taken collectively, these studies demonstrate a conserved role for TTF family members in diverse abiotic stress responses.

Cotton (*Gossypium hirsutum* L.) is an economically important textile fiber crop that is cultivated worldwide. Abiotic stress has become a major environmental factor limiting cotton cultivation and production due to global climate change and environmental pollution (Naeem et al., 2021; Saleem et al., 2021). Despite the economic ramifications of abiotic stress on cotton production, little is known about the mechanisms of abiotic stress tolerance in cotton compared to other agricultural crops. The obvious importance of the TTF family in abiotic stress responses in other crop species led us to hypothesize that this family of genes plays a similar role in cotton. We previously analyzed the expression of 24 cotton TTF genes in response to diverse abiotic stimuli to identify genes that were differentially expressed and involved in abiotic stress responses in cotton (Li et al., 2013). Of the 24 TFF genes analyzed, *GhGT23* was one of three that was differentially expressed in response to all of the abiotic stress treatments we tested. In this study, we further characterize the function of *GhGT23*, which belongs to the SIP subfamily of TFFs. The expression of *GhGT23* was analyzed in response to salt stress, drought stress, cold stress, and ABA treatment in cotton. Heterologous overexpression of *GhGT23* in *Arabidopsis* led to improved tolerance of salt and drought in seedlings, which was positively correlated with an increase in the expression of stress-related genes. Furthermore, GhGT23 could directly bind to multiple GT motifs and MYB elements. Our results further highlight the importance of TTF family members in plant abiotic stress responses and specifically provide key insights into the function of *GhGT23* in salt and drought tolerance in cotton.

## Materials and methods

### Plant material, growth conditions, and treatments

Seeds of the cotton (*G.hirsutum)* cultivar ‘Xinluzao 26’ were evenly distributed in a pot containing vermiculite and raised in a greenhouse at a temperature of 25 ± 2 °C with a 16-h/8-h light/dark cycle and a relative humidity of 65%. Fifteen days after planting, seedlings were treated with drought, NaCl, cold, or ABA (100 μmol/L) according to previously published methods (Li et al., 2022). Seedlings planted at the same time were also left untreated to serve as controls. Leaves were harvested at 1, 3, 6, and 12 h post-treatment. Tissue from roots, stems, and leaves of 15-day-old seedlings were collected for RNA isolation to assess tissue-specific gene expression. RNA was also isolated from flowers and ovules at 0 days post-anthesis (DPA) and cotton fibers at 12 DPA. All harvested tissue was immediately frozen in liquid nitrogen and stored at -70 °C for subsequent experiments. Each treatment was performed at least three independent times.

### Gene cloning and sequence analysis

Primers GhGT23F and GhGT23R were used to amplify the CDS of *GhGT23* from cotton leaf cDNA as described previously (Li et al., 2022). The amplified *GhGT23* fragment was subcloned into *pEASY-T1* (TransGen Biotech, Beijing, China), thereby creating *pEASY-T1::GhGT23*. Flanking BamHI and SalI restriction sites were then used to cut *GhGT23* from *pEASY-T1::GhGT23*, followed by ligation into the *pBI21*, *pBin438*, *pGAL4 DBD*, and *pGEX6p-1* expression vectors. The primers used for PCR are listed in **Table S1**. The ORF of *GhGT23* was translated using DNAStar, and ProtParam (http://web.expasy.org/cgi-bin/protparam/protparam) was used to estimate the protein molecular mass and isoelectric point. DNAMAN (V6.0) was used to align the GhGT23 protein sequence with homologs from *Gossypium hirsutum* and other species obtained from the NCBI database. Phylogenetic analyses were performed using MEGAX (http://www.megasoftware.net/) and ClustalX. SMART (http://smart.embl-heidelberg.de/) was used to predict conserved domains in GhGT23. Psort (http://www.psort.org/) was used to predict the subcellular localization of GhGT23.

### Subcellular localization of GhGT23

The cauliflower mosaic virus (CaMV) 35S promoter in *pBI221* (Chen et al., 2003) was used to drive the expression of *GhGT23* fused in-frame with the 3’ end of *GFP*. The resulting construct, *pBI221*::*GFP : GhGT23*, was transfected into *Arabidopsis* protoplasts by the PEG4000-mediated method described previously by Yoo et al. (2007). *Arabidopsis* protoplasts were isolated by following the previously published method by Wu et al. (2009). A Leica model TCS SP5 laser scanning confocal microscope (leica Microsystems CMS GmbH, Mannheim, Germany) was used to observe GFP fluorescence in protoplasts to determine the subcellular localization of GhGT23.

### Transformation of *Arabidopsis* plants

The expression plasmid *pBin::GhGT23* was transformed into *Agrobacterium* strain GV3101 using the electroporation method (Mersereau et al., 1990) and transformed into *Arabidopsis* using the floral dip method (Clough and Bent, 2010). Transgenic seedlings were screened for resistance to kanamycin (50 μg/mL) and cephamycin (25 μg/mL) on 1/2 MS agar medium, and gene insertion was confirmed by PCR. Three homozygous T3 plants with the highest transgene expression were kept for subsequent experiments.

### RNA extraction and quantitative real-time PCR

Total RNA was extracted from cotton plant tissues using the Biospin plant total RNA extraction kit (Bioer, Hangzhou, China). TRIzol reagent (Transgen, Beijing, China) was used to extract total RNA from transgenic *Arabidopsis* plants according to the manufacturer’s instructions. The quality and quantity of RNA were evaluated using a NanoDrop 1000 spectrophotometer (Thermo Fisher Scientific Inc., Waltham, MA, USA). RNA integrity was determined by electrophoresis on 1.2% agarose gels stained with ethidium bromide. First-strand cDNA was synthesized from total RNA (4 µg) using M-MLV reverse transcriptase and an oligo (dT) primer according to the manufacturer’s instructions (Promega, Madison, WI, USA). Relative gene expression levels were calculated using the 2^−ΔΔCT^ formula (Livak and Schmittgen, 2001). in triplicate using independent cDNA samples. All gene information and primers used in the qPCR analysis are listed in **Supplementary Table S1**.

### Transcriptional activation assay in *Arabidopsis* protoplasts

*GhGT23* was cloned in-frame with the DNA-binding domain of *Gal4* in *pGAL4 DBD* to create *pGAL4 DBD : GhGT23* (Hao et al., 2010). Plasmid *pGAL4 DBD : GhGT23* was co-transfected with reporter *GAL4::Luc* into *Arabidopsis* protoplasts by PEG-mediated transformation. Luminescence from luciferase was detected using the Dual-Luciferase Reporter^®^ assay system (Promega) and GloMax 20/20 microplate luminometer (Promega). The VP16 activation domain in *pBD* was used as a positive control, and *GAL4 DBD* was used as a negative control.

### Electrophoretic mobility shift assay (EMSA)

**Figure 1:**
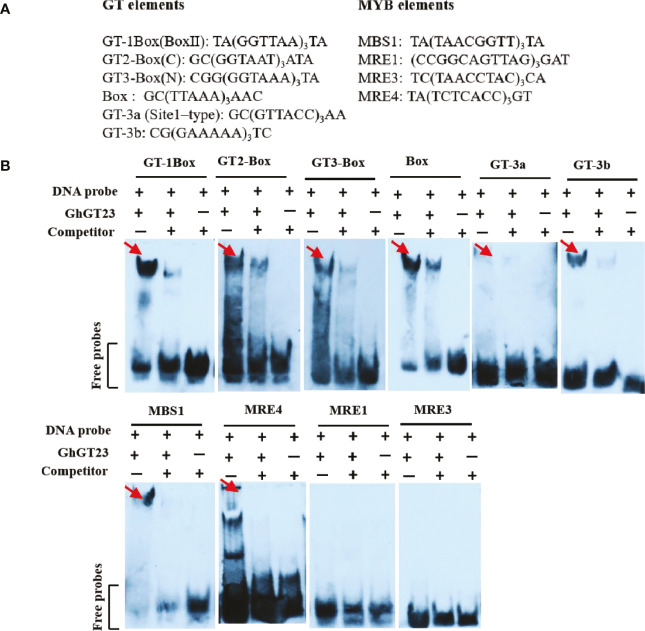
*In vitro* DNA-binding activity of GhGT23. **(A)** Sequences of the GT and MYB elements used in the electrophoretic mobility shift assay (EMSA) are shown. **(B)** Recombinant GhGT23 protein was incubated with the DNA probes listed in **(A)** in the presence or absence of a competitor. The red arrows indicate the band corresponding to the GhGT23/DNA complex.

*GhGT23* was cloned in-frame with the glutathione S-transferase (GST) tag in *pGEX6p-1*. The resulting plasmid was then transformed into *Escherichia coli* BL21 (DE3) cells to express the GST-GhGT23 fusion protein. The GST-GhGT23 protein was purified by glutathione Sepharose 4B affinity chromatography (GE Healthcare, Chicago, IL, USA). All double-stranded DNA fragments used in the EMSA were synthesized as single-stranded oligonucleotides in complementary pairs by TakaRa (Da Lian). Single-stranded oligonucleotides were annealed to form double-stranded DNA by mixing in equal ratios (4 μM each) in 50 mM NaCl buffer, heating to 70 °C for 5 min, and slowly cooling to room temperature. The EMSA was performed using digoxigenin-labeled probes as previously described, whereas the competitor control consisted of double-stranded DNA without digoxigenin-labeling (Song et al., 2013). All oligonucleotide sequences are presented in **Figure 1A**.

### Analysis of transgenic plants under salt and drought conditions

Seeds from the three homozygous T3 lines overexpressing *GhGT23* (L24, L29, and L36) and *Arabidopsis* ecotype Columbia (WT) were used for phenotypic analyses. For salt stress assays using green seedlings, seeds were plated on 1/2 MS medium containing 150 mM NaCl. The plates were stratified at 4°C for 3 days before being placed in a growth chamber set to 23°C with a 16-h/8-h light/dark cycle. The effects on seedling morphogenesis were observed after 16 days of growth, and the percentage of green seedlings was determined. For salt treatment, 12-day-old seedlings were transferred into 100% vermiculite saturated with 200 mM NaCl. After 12 days, seedlings were transferred into vermiculite without NaCl to recover from the salt stress. The survival rates of WT and transgenic plants were recorded after three days of recovery. Drought treatment was conducted by transferring 5-day-old seedlings to 1/2 MS medium supplemented with 300 mM mannitol. Treated and non-treated seedlings were transferred to vermiculite after 20 days to recover before determining the plant survival rate. Drought stress was administered by withholding water from 12-day-old seedlings for 30 days. Images of the plants were taken, and the survival rate was calculated, after re-watering for 3 days.

Root growth in response to salt and drought stress was assessed by germinating seeds on 1/2 MS medium and transferring 4-day-old seedlings to 1/2 MS medium supplemented with 135 mM NaCl or 300 mM mannitol. Seedlings were placed vertically in a growth chamber set to 23°C with a 16-h/8-h light/dark cycle. Images of the plants were taken after 15 days, and the primary root length was measured. Each sample contained eight seedlings, and the experiments were repeated three times with consistent results.

### Statistical analysis

Microsoft Excel 2020 and SPSS v18.0 software were used for data analysis. Differences between groups were tested using one-way analysis of variance (ANOVA), followed by Duncan’s multiple comparison test at p < 0.05.

## Results

### Phylogenetic and sequence analysis of *GhGT23*

**Figure 2:**
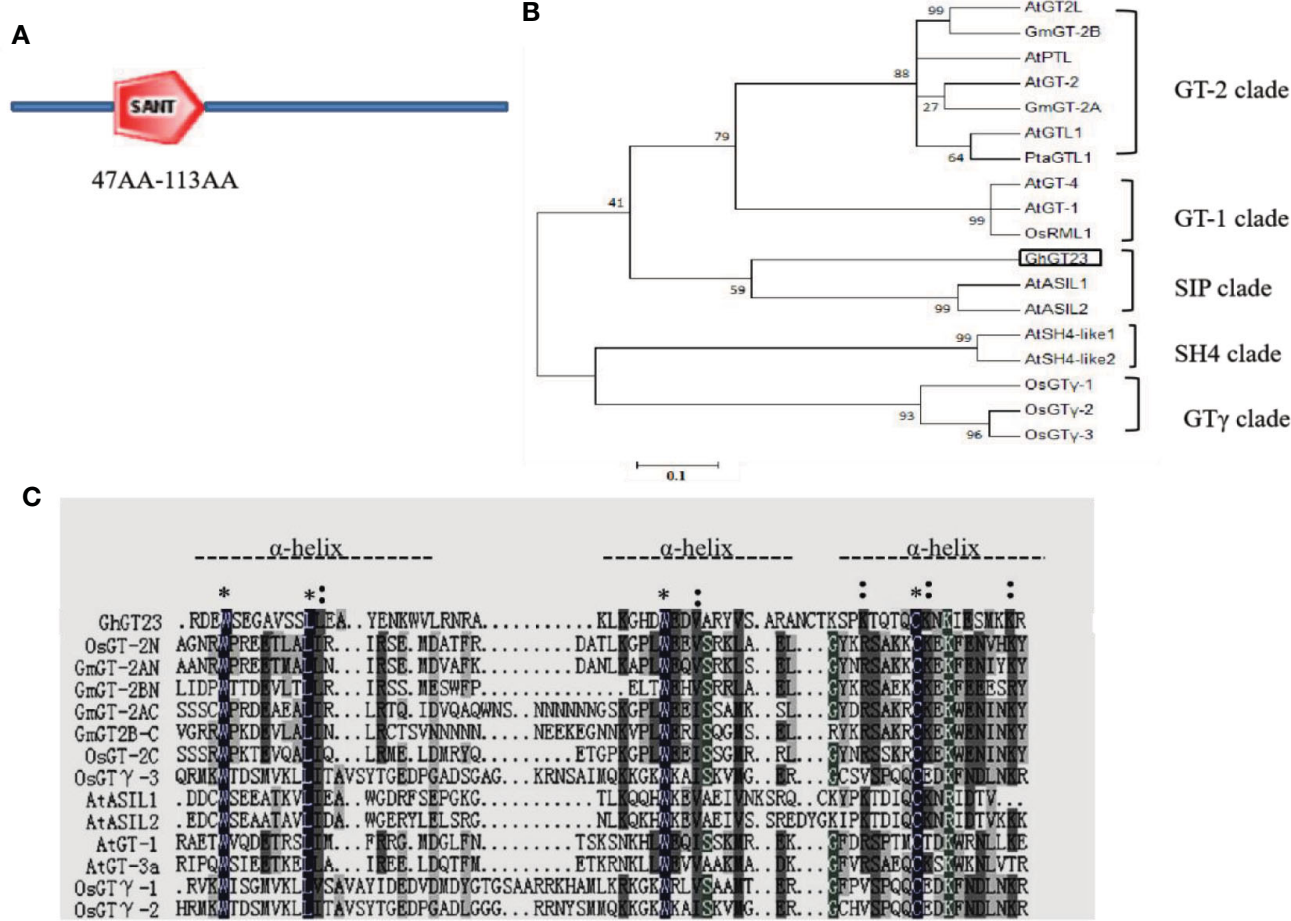
Schematic representation, phylogenetic analysis, and amino acid sequence alignment of GhGT23. **(A)** Schematic diagram of the GhGT23 protein with the SANT domain highlighted between residues 47 and 113. **(B)** Phylogenetic analysis of GhGT23 with other TTFs. The analysis was performed using MEGA 6.0 with the neighbor joining method and 1000 replicates. Numbers on the figure are bootstrap values. The sequences are from rice (*Oryza sativa*), soybean (*Glycine max*), and *Arabidopsis* (*Arabidopsis thaliana*) plants. GT-1 clade: AtGT-1 (At1g13450), AtGT-4 (At3g25990), OsRML1 (AL627350); GT-2 clade: AtGT-2 (At1g76890), AtGTL1 (At1g33240), AtGT2L (At5g28300), AtPTL (At5g03680), GmGT-2A (EF221753), GmGT-2B (EF221754), PtaGTL1 (JN113092); SH4 clade: AtSH4-like1 (At2g35640), AtSH4-like2 (At1g31310); GT clade: OsGT-1 (Os02g33770), OsGT-2 (Os11g06410), OsGT-3 (Os12g06640); and SIP clade: AtASIL1 (At1g54060), AtASIL2(At3g14180), GhGT23. **(C)** Multiple sequence alignment of the SANT domain from the TTFs in **(B)**. * indicates a completely conserved amino acid residue; indicates a partially conserved amino acid. The dotted line denotes helices in the trihelix DNA-binding domain.

*GhGT23* (GenBank accession number: JQ013095) was PCR-amplified from a cDNA library created from RNA collected from cotton cotyledons. The coding sequence (CDS) lengths of *GhGT23* were 1125 bp. The GhGT23 protein consisted of 375 amino acids, with a molecular weight of 40.90 kDa and an isoelectric point of 9.42. SMART analysis revealed the presence of one SANT (Swi3, Ada2, N-Cor, and TFIIB) domain between amino acids 47 and 113 of GhGT23 (**Figure 2A**). A phylogenetic tree was constructed using GhGT23 and its homologs from cotton, soybean, *Arabidopsis*, and rice. The phylogenetic tree revealed that GhGT23 falls into the SIP subfamily of TTFs and is most closely related to *Arabidopsis* AtASIL1 and AtASIL1 (**Figure 2B**). Multiple sequence alignment between the same proteins used in the phylogenetic analysis confirmed that the SANT domain of GhGT23 is rich in basic, acidic, and proline residues within the tandem helices (**Figure 2C**). The conserved residues that were part of the tryptophan repeat within the tandem helices consisted of W-L-W-E-V, which is expected for SIP subfamily members. These results suggest that GhGT23 is a member of the SIP subfamily of TTFs.

### *GhGT23* is highly expressed in cotton fibers and is influenced by abiotic stress

**Figure 3:**
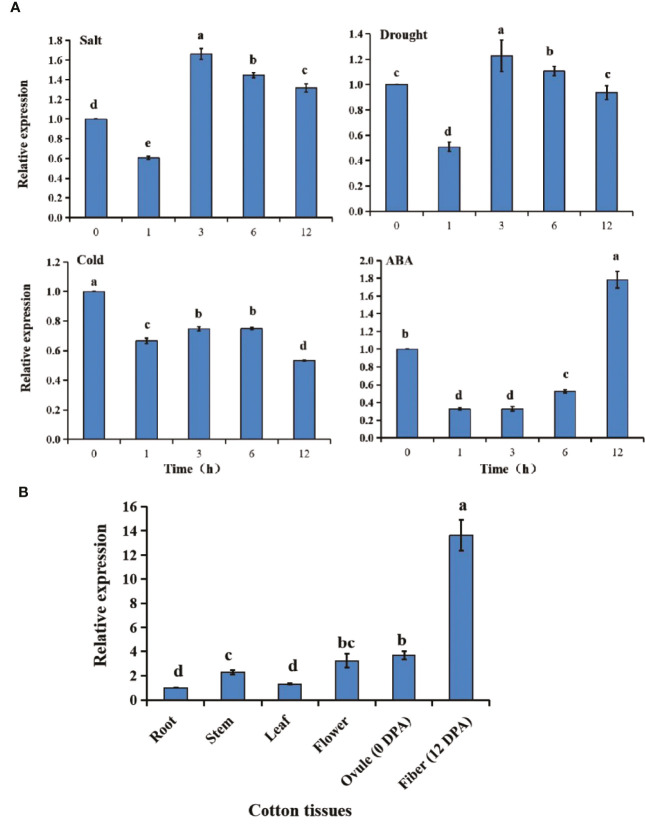
The expression of the *GhGT23* gene in response to different stresses and ABA treatment as well as in different tissue types. **(A)** Expression of *GhGT23* in cotton seedlings in response to ABA treatment and different stresses. The 0-h expression level was artificially set to 1. **(B)** *GhGT23* expression in various cotton plant tissues. The expression of *GhGT23* in the root was artificially set to 1. DPA, days post-anthesis. Different letters indicate significant differences (p < 0.05) determined using ANOVA, followed by Duncan’s multiple comparison test.

*GhGT23* expression was monitored over time using qRT-PCR in cotton plants subjected to salt stress, drought stress, cold stress, and ABA treatment. The results were shown in **Figure 3A**, for all treatments, *GhGT23* expression decreased within the first hour after the treatment was administered. Plants subjected to salt and drought stress had peak *GhGT23* expression at 3 h, followed by a gradual decline. *GhGT23* expression remained relatively unchanged in plants exposed to 1 h of cold stress, which never reached the 0-h expression level. Plants treated with ABA recovered the *GhGT23* expression level and even exceeded the 0-h expression level at 12 h. Although *GhGT23* was expressed in all cotton plant tissues, it was substantially higher in cotton fibers at 12 DPA than in the other tissues sampled (**Figure 3B**).

### GhGT23 is localized to the nucleus

**Figure 4:**
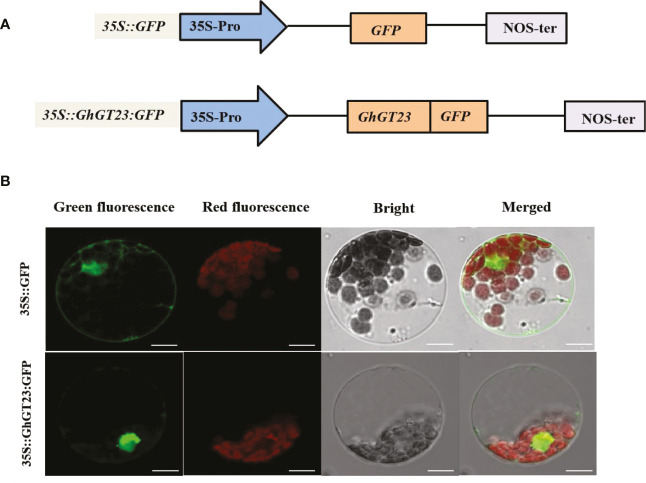
The subcellular localization of GhGT23 in *Arabidopsis* protoplasts. **(A)** Diagram of the GFP-fusion vector (*35S::GhGT23:GFP*) and control construct (*35S::GFP*) used in *Arabidopsis* protoplast transfections. **(B)** Transient expression of *35S::GhGT23:GFP* and free *35S::GFP* in *Arabidopsis* protoplasts. Green fluorescence from free GFP and GhGT23:GFP protein accumulation can be seen in the nucleus and cytoplasm of protoplasts. Chlorophyll autofluorescence is shown in red, and the periphery of the protoplasts can be seen in the brightfield images. Images were taken using a laser scanning confocal microscope with the following filters: GFP (excitation 488 nm; emission 509 nm) and chlorophyll autofluorescence (excitation 448 nm; emission 647 nm). Bars = 10 μm.

The subcellular localization for GhGT23 was predicted using Psort (http://www.psort.org/), which was anticipated as it is a TTF family member. The subcellular localization was confirmed by fusing *GhGT23* to the 5’-end of *GFP* in the *pBI221* vector and using confocal laser scanning microscopy to observe GFP fluorescence in *Arabidopsis* protoplasts (**Figure 4A**). Approximately 17 h post-transfection, green fluorescence was observed in the nucleus and cytoplasm of protoplasts expressing the free *GFP* control, whereas fluorescence in protoplasts expressing *GhGT23:GFP* was restricted to the nucleus (**Figure 4B**). These results indicate that GhGT23 is localized to the nucleus, which is consistent with the prediction from Psort and its predicted role as a TTF.

### *GhGT23* overexpression confers enhanced salt stress tolerance in *Arabidopsis*

**Figure 5:**
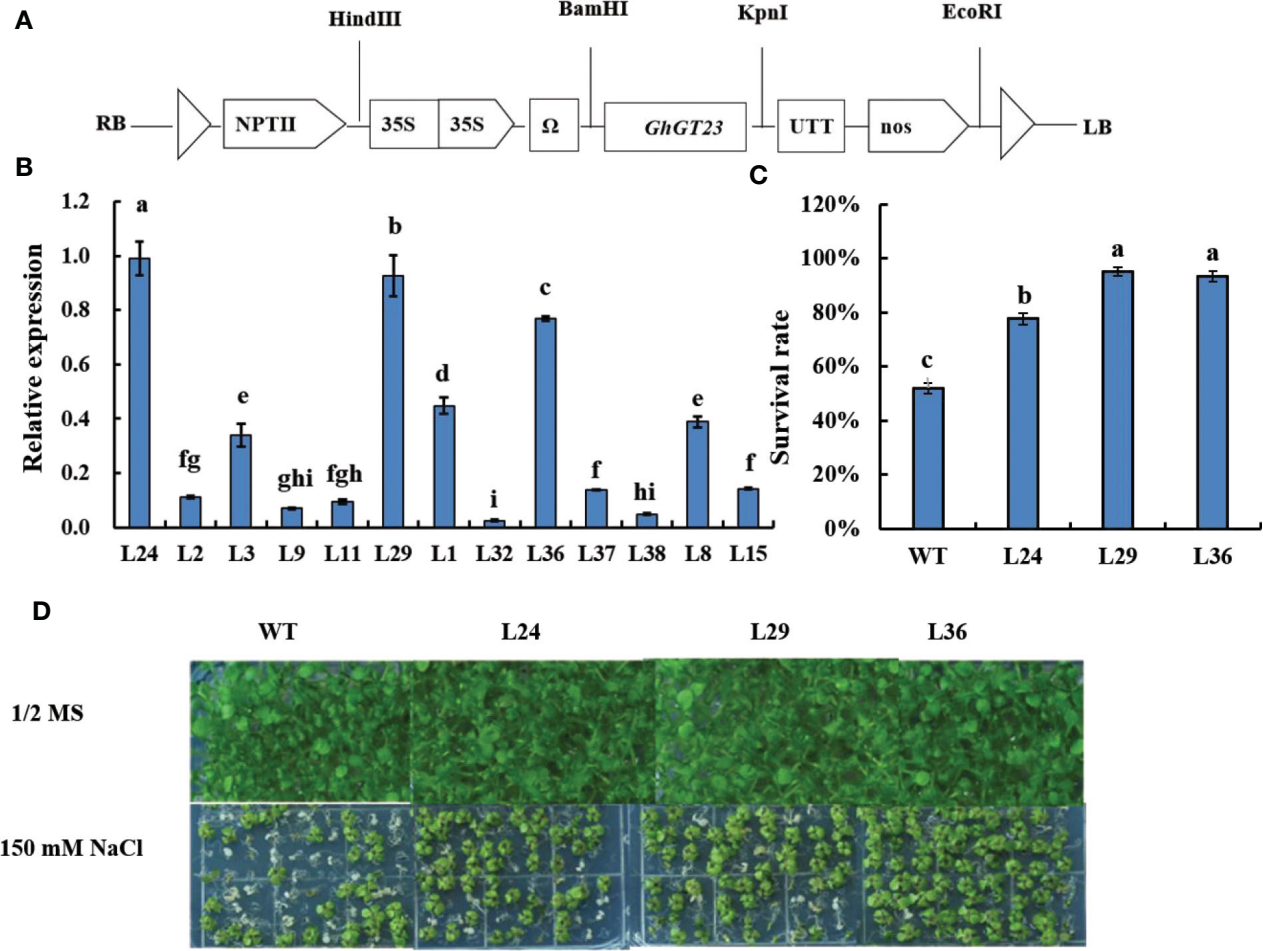
The survival rate of *Arabidopsis GhGT23*-overexpressing lines exposed to 150 mM NaCl on 1/2 MS agar plates. **(A)** Diagram of the *GhGT23*-overexpression cassette. **(B)** Quantification of *GhGT23* transgene expression in *Arabidopsis* transgenic lines. **(C)** Quantification of the seedling survival rate in WT and transgenic lines after 16 days of growth on 1/2 MS agar supplemented with 150 mM NaCl. Error bars indicate standard deviation and different letters indicate significant differences (p < 0.05), which were determined using ANOVA, followed by Duncan’s multiple comparison test. **(D)** Images of WT and transgenic lines taken after 16 days of growth on 1/2 MS agar with and without 150 mM NaCl.

Three independent T3 transgenic *Arabidopsis* lines with the highest expression of *GhGT23* (L24, L29, and L36) were subjected to 150 mM NaCl to determine if *GhGT23* overexpression could enhance salt stress tolerance (**Figures 5A, B**). Seeds from WT, L24, L29, and L36 plants were sown on 1/2 MS agar plates with and without 150 mM NaCl. After 16 days of growth, plants were phenotyped and green seedlings were counted. No differences in growth were observed between WT and transgenic plants in the absence of NaCl. However, transgenic seedlings were much greener than WT seedlings in the presence of 150 mM NaCl (**Figure 5C**). The percentage of seedlings that were still green following salt stress were 77.78%, 95.19%, 93.46%, and 52.10% in L24, L29, L36, and WT lines, respectively (**Figure 5D**).

**Figure 6:**
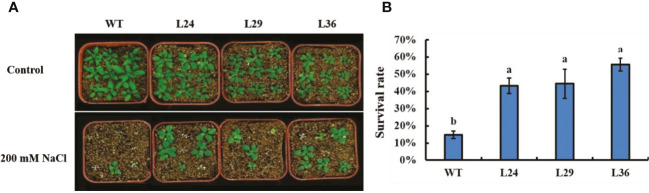
The survival rate of *Arabidopsis GhGT23*-overexpressing lines exposed to 200 mM NaCl in vermiculite. **(A)** Images of WT and transgenic lines taken after 12 days of growth in vermiculite with and without 200 mM NaCl. **(B)** Quantification of the survival rates of WT and transgenic lines in panel **(A)**. Each bar represents the average of three experiments with 36 plants used in each experiment. Error bars indicate standard deviation and different letters indicate significant differences (p < 0.05) which were determined using ANOVA, followed by Duncan’s multiple comparison test.

To better replicate real-world salt stress, 12-day-old seedlings were transplanted into vermiculite saturated with 200 mM NaCl. Seedlings were removed after 12 days and transplanted in soil lacking NaCl to recover. The survival rate of WT and transgenic plants was assessed after a 3-day recovery period. No significant difference was observed between WT and transgenic plants under normal conditions (**Figure 6A**). However, the survival rates of the transgenic lines were approximately 3-4 times longer than the WT line when plants were treated with 200 mM NaCl (**Figure 6B**). Taken collectively, these results demonstrate that *GhGT23* overexpression in *Arabidopsis* conferred enhanced salt stress tolerance *in vitro* and *in vivo*.

### *GhGT23* overexpression confers enhanced drought tolerance in *Arabidopsis*

**Figure 7:**
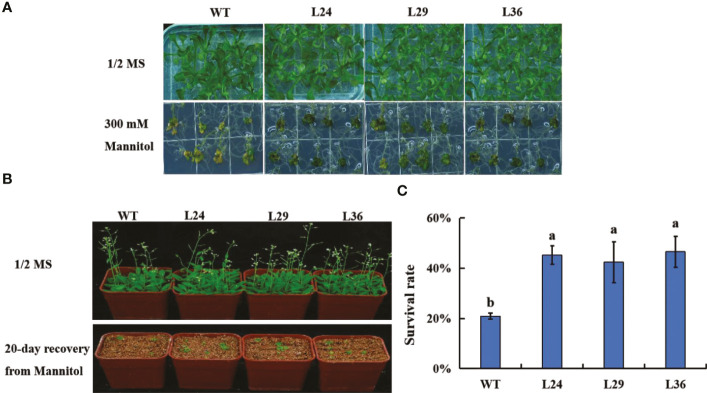
The effect of *GhGT23* overexpression on *Arabidopsis* in response to drought-simulating mannitol treatment. **(A)** Images of WT and transgenic *Arabidopsis* plants overexpressing *GhGT23* (L24, L29, and L36) 12-days after being transferred to 1/2 MS agar with and without 300 mM mannitol. **(B)** Images of WT and transgenic *Arabidopsis* plants treated with 300 mM mannitol, followed by a 20-day recovery period in vermiculite. **(C)** Survival rates of WT and transgenic plants after a 20-day recovery period from mannitol treatment. Each bar represents the average of three experiments with 36 plants used in each experiment. Error bars indicate standard deviation and different letters indicate significant differences (p < 0.05), which were determined using ANOVA, followed by Duncan’s multiple comparison test.

Drought tolerance in the *GhGT23* transgenic lines was assessed by plating 5-day-old seedlings on 1/2 MS agar with and without 300 mM mannitol. No differences in growth were observed between WT and transgenic plants in the absence of mannitol. After 20 days of growth on media containing mannitol, WT and transgenic plants were smaller with brown and yellow leaves compared to the non-treated plants. These phenotypes were more severe in WT plants (**Figure 7A**). Treated and non-treated seedlings were transferred into pots containing vermiculite without mannitol to allow surviving plants to recover (**Figure 7B**). The survival rate of the WT and transgenic lines was assessed after a 20-day recovery period. Wild-type *Arabidopsis* plants had a 20.83% survival rate, whereas L24, L29, and L36 had survival rates of 45.14%, 42.36%, and 46.53%, respectively (**Figure 7C**). These results indicate that overexpression of *GhGT23* in *Arabidopsis* confers enhanced drought stress tolerance.

**Figure 8:**
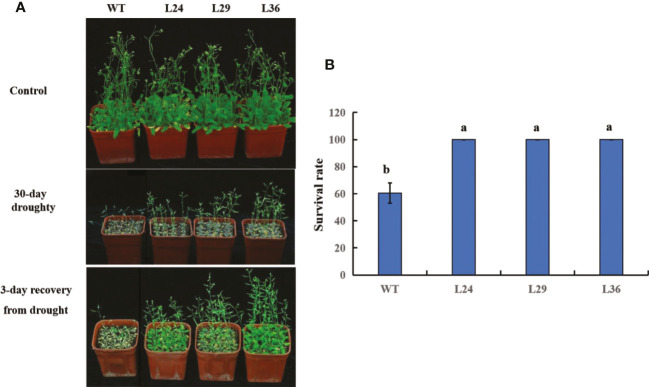
The effect of *GhGT23* overexpression on *Arabidopsis* in response to 30-day drought treatment. **(A)** Images of WT and transgenic *Arabidopsis* plants overexpressing *GhGT23* (L24, L29, and L36) 30 days after a normal watering schedule (control) and drought. Plants were also imaged 3 days after being watered at the end of the drought period. **(B)** Survival rates of WT and transgenic plants after a 3-day recovery period from drought treatment. Each bar represents the average of three experiments with 64 plants used in each experiment. Error bars indicate standard deviation and different letters indicate significant differences (p < 0.05), which were determined using ANOVA, followed by Duncan’s multiple comparison test.

To better simulate real-world drought stress, 12-day-old seedlings were not watered for 30 days. At the end of the 30-day drought period, plants were watered, and images were taken after 3 days to assess plant survival. No difference between the WT and transgenic plants was observed in the absence of drought, but all plants were severely dwarfed and wilted at the end of the 30-day drought period (**Figure 8A**). However, these phenotypes were less severe in the transgenic lines, and 3 days after watering, the transgenic lines completely recovered, while only 60% of the WT plants survived (**Figures 8A, B**). These results show that overexpressing the cotton *GhGT23* gene in transgenic *Arabidopsis* plants increases drought tolerance.

### *GhGT23* overexpression sustains root growth in *Arabidopsis* upon salt and drought stress

**Figure 9:**
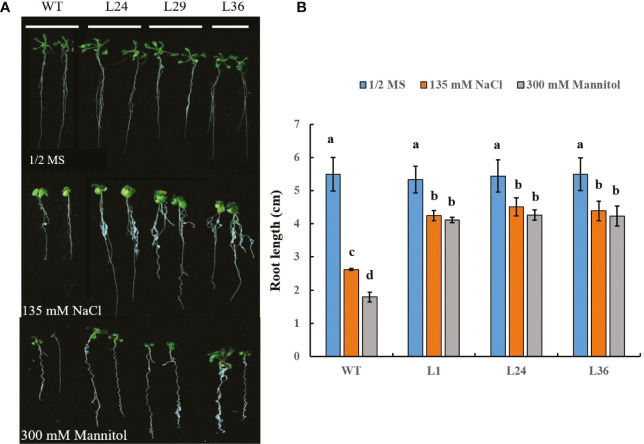
The effects of salt and mannitol stress on root length in *Arabidopsis GhGT23*-overexpressing lines. **(A)** Root images from plants growing on 1/2 MS agar, 1/2 MS agar supplemented with 135 mM NaCl, and 1/2 MS agar supplemented with 300 mM mannitol. **(B)** Quantification of primary root lengths from plants growing on 1/2 MS agar with and without 135 mM NaCl and 300 mM mannitol. Each bar represents the average of three experiments with 24 plants used in each experiment. Error bars indicate standard deviation and different letters indicate significant differences (p < 0.05) which were determined using ANOVA, followed by Duncan’s multiple comparison test. Each data point is the average of three experiments, with each experiment consisting of 48 plants.

In addition to the effects of salt and drought stress on shoot development, these stresses can also adversely affect root development. We sought to determine if the roots of transgenic *GhGT23*-expressing *Arabidopsis* seedlings were more resistant to NaCl and mannitol than WT plants. In the absence of either stress, the root lengths of WT and transgenic plants were the same, but when plants were raised on 1/2 MS agar containing 135 mM NaCl or 300 mM mannitol the root lengths of all lines decreased significantly (**Figure 9**). However, root length reduction in the presence of these stresses was significantly less severe in the transgenic plants (**Figure 9**). These results largely mirror the effects of salt and drought stress on shoots and provide additional evidence that overexpression of *GhGT23* in *Arabidopsis* confers enhanced tolerance against these stresses.

### Stress-responsive genes are upregulated in *GhGT23*-transgenic plants

**Figure 10:**
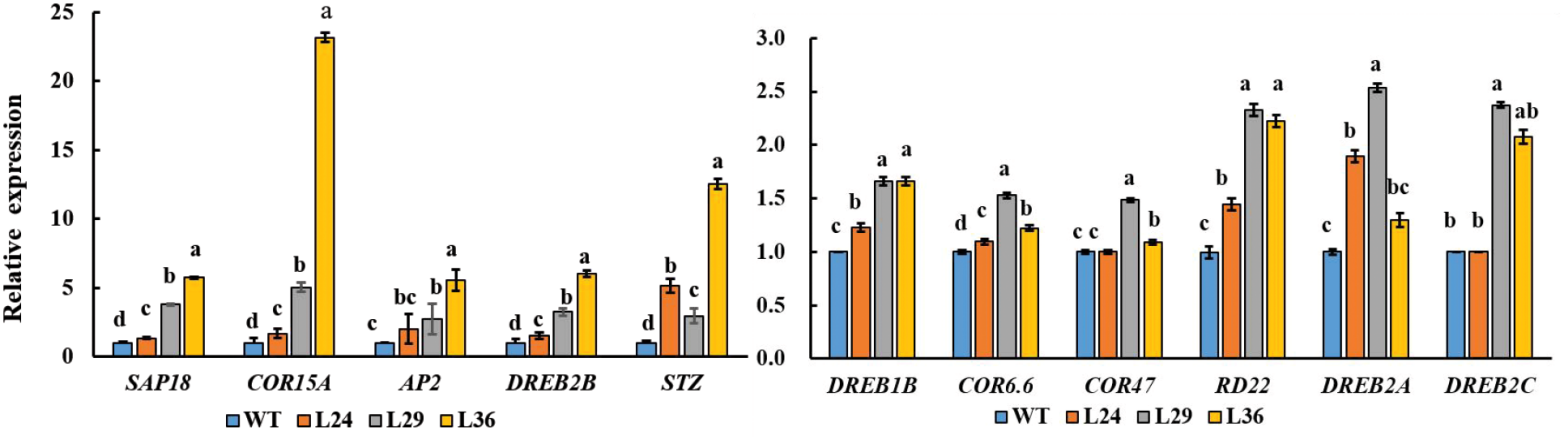
The expression of stress-related genes in WT and transgenic *Arabidopsis* lines overexpressing *GhGT23*. Each bar represents the average of three experiments with 12 plants used in each experiment. The expression of stress-related genes in WT plant was set to 1 as control. Different letters indicate significant differences between the control plants and transgenic lines from the same gene which were determined using ANOVA, followed by Duncan’s multiple comparison test (p < 0.05).

Plants have evolved a variety of regulatory mechanisms for drought adaptation, and the regulation of hormones, especially abscisic acid (ABA), is one of the most important strategies (Drake et al., 2013) When plants are subjected to drought stress, ABA levels increase with the severity of drought stress (Bhusal et al., 2019). We hypothesized that *GhGT23* may promote salt and drought tolerance in transgenic *Arabidopsis* plants by regulating genes involved in plant stress responses. To test this hypothesis, we analyzed the expression levels of ABA-responsive, cold and drought stress-tolerance-related genes.The expression levels of 11 abiotic stress-responsive genes: *DREB1B*, *COR6.6*, *COR47*, *RD22*, SAP18, *COR15A*, *DREB2A*, *DREB2B, STZ*, *AP2*, and *DREB2C* were higher in transgenic plants overexpressing *GhGT23* than in WT plants (**Figure 10**).

### GhGT23 has no transcriptional activation activity as revealed by dual-luciferase assay

**Figure 11:**
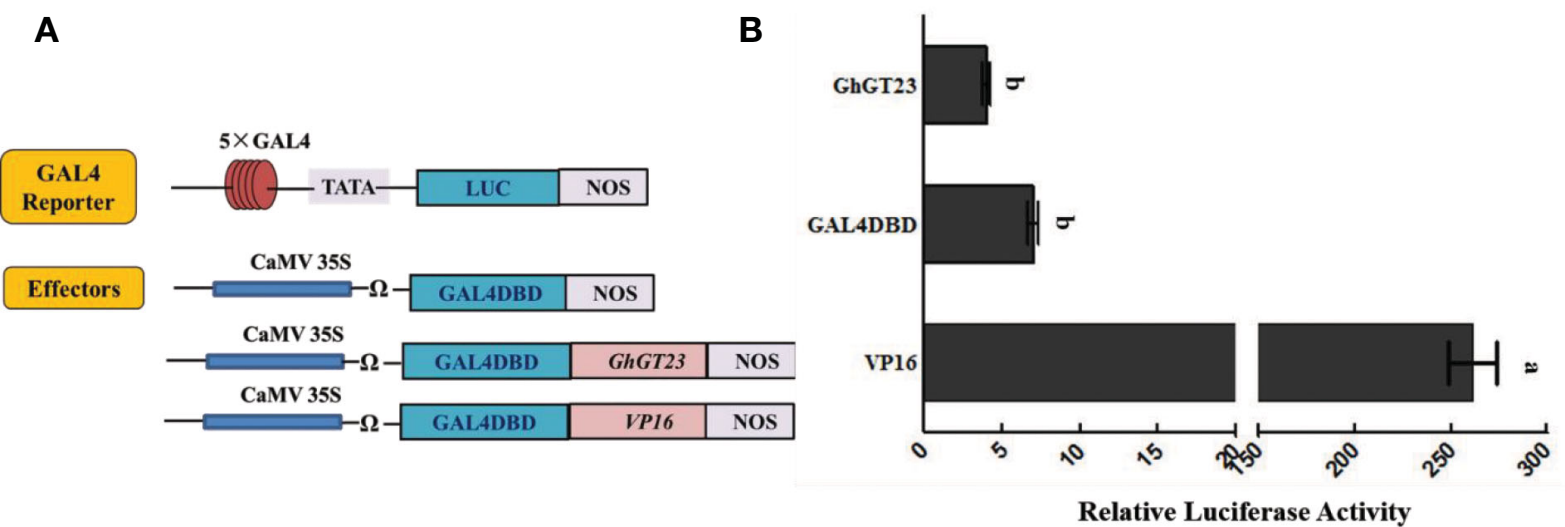
Assessing the transcriptional activity of *GhGT23* with a dual-luciferase reporter assay. **(A)** Diagram of the reporter and effector constructs used in the dual-luciferase reporter assay to assess the transcriptional activity of *GhGT23*. The GAL4 reporter was co-transfected with Renilla luciferase and each of the three effectors. **(B)** Luminescence from luciferase was quantified to assess the transcriptional activity of *GhGT23*. Each bar represents the average of three experiments. Error bars indicate standard deviation and different letters indicate significant differences (p < 0.05) which were determined using ANOVA, followed by Duncan’s multiple comparison test.

Transcription factors can activate or repress the expression of the genes that they regulate. To determine if GhGT23 can activate transcription, we performed a dual-luciferase reporter assay in *Arabidopsis* protoplasts. Firefly *luciferase* expression was regulated by a promoter consisting of five tandem GAL4 binding sites and the minimal TATA region of the cauliflower mosaic virus (CaMV) 35S promoter (**Figure 11A**). *GhGT23* was expressed downstream and in-frame with the yeast *GAL4 DNA-binding domain* (*GAL4DBD*) and expression of the *GhGT23:GAL4DBD* fusion gene was driven by a *35S* promoter (**Figure 11A**). Expression of *GAL4DBD* alone served as a negative control, whereas *VP16* served as a positive control for the activation of luciferase expression. Renilla luciferase expression driven by a 35S promoter served as an internal control. The GAL4 reporter vector and Renilla luciferase internal control vector were co-transfected with each of the three effector vectors into *Arabidopsis* protoplasts. Interestingly, we found that *GhGT23:GAL4DBD* expression did not activate luciferase expression, suggesting that GhGT23 is a TTF that may lack transcriptional activation activity (**Figure 11B**).

### GhGT23 binds GT and MYB promoter elements *in vitro*

TTFs bind GT cis-elements in the promoters of target genes to activate or repress gene expression. Variations exist in the sequences of GT elements, and each TTF subfamily has a preference for the specific GT element that they bind. The GT-1 and GT-3 subfamilies have a single DNA-binding domain that specifically binds the Box II element (5’-GTGTGGTTAATATG-3’) and 5’-GTTAC-3’ motif. Members of the GT-2 subfamily have two DNA-binding domains that can bind to GT2-Box (5’-GCGGTAATTAA-3’) and GT3-Box (5’-GAGGTAAATCCGCGA-3’) elements (Xie et al., 2009; Kaplan-Levy et al., 2012). An electrophoretic mobility shift assay (EMSA) was performed to determine if GhGT23 could bind to several known GT and MYB elements (**Figure 1A**). We found that the GhGT23 protein could bind to the GT elements GT-1Box (Box II), GT2-Box, GT3-Box, Box, GT-3a (Site1-type), and GT-3b and the MYB elements MBS1 and MRE4 (**Figure 1B**). Although GhGT23 could bind to every GT element and two of the MYB elements tested, we did not observe binding to the MYB elements MRE1 and MRE3. These results are interesting because they suggest that GhGT23 is more promiscuous than most TTFs in its ability to bind diverse GT and MYB elements.

## Discussion

Due to the reduction of arable land worldwide, cotton production has had to compete with grain production over shrinking sources of quality farmland. One strategy for the sustained production of cotton is to develop varieties that can grow in saline-alkali soil that is unsuitable for grain production. Current research efforts are focused on improving abiotic stress tolerance in cotton through genetic engineering of key regulators in stress response pathways. Common targets for engineering are key transcription factors that regulate the expression of downstream stress-response genes (Liu et al., 2022; Zhang et al., 2022). Recent studies of TTF family members in diverse plant species have revealed that these transcription factors are involved in plant responses to abiotic stresses such as salt and drought. A previous study by our lab identified *GhGT23* as a TTF in cotton that is differentially expressed in response to multiple abiotic stresses (Li et al., 2013). In this study, we further characterize the function of *GhGT23* in salt and drought stress in transgenic *Arabidopsis* lines overexpressing *GhGT23*.

*GhGT23* was cloned from upland cotton, and a SMART analysis of the protein sequence identified a single SANT domain (**Figure 2A**). The SANT domain is multifunctional and participates in both protein-protein and protein-DNA interactions; it is closely related to enzymatic activity and substrate affinity (Boyer et al., 2002; Boyer et al., 2004; Engel et al., 2018). Phylogenetic analysis revealed that GhGT23 falls within the SIP subfamily of TTFs along with *Arabidopsis* AtASIL1 and AtASIL2 (**Figure 2B**). AtSIL1 and AtSIL2 are involved in stem cell regulation, embryo patterning, and the transition from vegetative to reproductive growth in *Arabidopsis*, but no role in abiotic stress tolerance has been described for these TTFs (Willmann et al., 2011; Luo et al., 2012). Members of other TTF subfamilies, such as *Arabidopsis AST1* and *Brassica napus BnSIP1-1*, do regulate the expression of downstream genes that promote enhanced tolerance to salt, osmotic, and drought stress (Luo et al., 2017; Xu et al., 2018). We demonstrated that *GhGT23* expression was affected by salt, drought, cold, and ABA treatments, and *GhGT23* was highly expressed under non-stressed conditions in cotton fibers (**Figures 3A, B**). The nuclear localization of GhGT23 we observed in *Arabidopsis* protoplasts provided additional evidence that this is a novel cotton TTF in the SIP subfamily that may regulate abiotic stress responses through an ABA-dependent signaling pathway (**Figure 4**).

Generating stable transgenic cotton plants is challenging due to cost, time, and technical difficulties. As a cheaper, quicker, and easier alternative, TTFs from soybean and poplar have been successfully expressed in *Arabidopsis* to study their role in abiotic stress responses (Xie et al., 2009; Weng et al., 2012). We generated stable transgenic *Arabidopsis* lines overexpressing *GhGT23* to gain further insight into the biological function of this TTF. These transgenic *Arabidopsis* lines had enhanced above-ground tolerance to salt and drought stress in both *in vivo* and *in vitro* assays (**Figures 5**–**8**). Additionally, the primary roots of *Arabidopsis* expressing *GhGT23* were longer than those of WT plants upon salt and drought stress (**Figure 9**). These results demonstrate that overexpression of *GhGT23* confers enhanced salt and drought stress tolerance in a heterologous system.

To gain further insight into the molecular mechanism underlying improved salt and drought tolerance in the *GhGT23*-overexpressing *Arabidopsis* lines, we selected some stress marker genes in *Arabidopsis* and monitored their expression in WT and transgenic lines after 7-day salt exposure. We noticed a significant increase in the expression of *DREB1B*, *COR6.6*, *COR47*, *RD22*, *SAP18*, *COR15A*, *DREB2A*, *DREB2B*, *STZ*, *AP2*, and *DREB2C* (**Figure 10**). Dehydration-responsive element-binding (DREB) proteins are transcription factors involved in cold, drought, and salt stress response pathways (Maruyama et al., 2004; Lee et al., 2005; Oh et al., 2005; Matsukura et al., 2010; Lei et al., 2016). The *Arabidopsis* DREB-like transcription factors have been shown to regulate the expression of downstream stress-related genes including *HsfA3*, *rd29A*/*cor78*, *kin1*, *kin2*, *cor6.6*/*kin2*, *cor15a*, *cor47/rd17*, and *erd10* to improve the tolerance of multiple stresses in transgenic plants (Liu et al., 1998; Seki et al., 2001; Maruyama et al., 2004; Zhao et al., 2007). Part of their function is to regulate the expression of cold-responsive genes that are involved in stress tolerance. *AtSTZ* is a positive regulator of salt stress tolerance, whereas *AtSAP18* is a negative regulator of drought and salt stress tolerance in *Arabidopsis* (Sakamoto et al., 2000; Sakamoto et al., 2004). These results demonstrate that overexpression of *GhGT23* in *Arabidopsis* broadly affects the expression of many genes involved in plant abiotic stress tolerance.

The results of the dual-luciferase reporter assay indicated that GhGT23 does not have the ability to activate transcription on its own in *Arabidopsis* protoplasts (**Figure 11**). However, our EMSA results demonstrated that GhGT23 can bind all six of the GT elements tested and two out of four MYB elements tested (**Figure 1**). This was an unexpected discovery because TTFs are typically very selective for the cis-elements that they bind. These results also raise an interesting question regarding the function of GhGT23. If GhGT23 is not a transcriptional activator, what purpose does it have in binding GT and MYB elements? It is possible that GhGT23 is a transcriptional repressor or perhaps a transcriptional activator that requires other proteins in cotton that are not conserved in *Arabidopsis*.

## Conclusions

In this study, we cloned and characterized *GhGT23*, a novel TTF in cotton and a member of the SIP subfamily. We observed that the expression of *GhGT23* is influenced by drought, salt, cold, and ABA treatments. Remarkably, *Arabidopsis* transgenic lines overexpressing *GhGT23* exhibit enhanced tolerance of salt and drought stress, which results in better above-ground and below-ground growth in the presence of these stresses. We also found that *GhGT23* overexpression increases the expression of multiple downstream stress-related genes in *Arabidopsis*, and the GhGT23 protein binds to diverse GT and MYB cis-elements. Interestingly, GhGT23 does not exhibit transcriptional activation activity, which brings into question the biological significance of GhGT23 binding to GT and MYB cis-elements. It is possible that GhGT23 functions as a transcriptional repressor or requires post-translational modifications performed by proteins in cotton that are not conserved in *Arabidopsis*. Future studies addressing these questions will reveal more about the molecular mechanism through which GhGT23, and possibly other SIP subfamily members, regulate plant stress responses.

## Data availability statement

The raw sequence data reported in this study have been deposited into the National Center for Biotechnology Information Sequence Read Archive (GhGT23 GenBank accession number: JQ013095).

## Author contributions

YL and ZX designed the research. YL, ZH, and YD performed the research. YL and ZX analyzed the data. YL wrote the paper and ZX supervised the project. All authors contributed to the article and approved the submitted version.
