# Supplementary material for: Overexpression of the cotton trihelix transcription factor GhGT23 in Arabidopsis mediates salt and drought stress tolerance by binding to GT and MYB promoter elements in stress-related genes
Source: Front Plant Sci. 2023 Mar 2;14:1144650. doi: 10.3389/fpls.2023.1144650 (PMC10017854; doi:10.3389/fpls.2023.1144650)
Supplement: Supplementary file 1 [file Table_1.docx]

**Overexpression of the cotton trihelix transcription factor *GhGT23* in *Arabidopsis* mediates salt and drought stress tolerance by binding to GT and MYB promoter elements in stress-related genes**

**Supplementary Data**

Supplementary Table 1

Primers used in this study

| Primer’s name | Primer’s sequence (5’-3’) |
| --- | --- |
| GhGT23F(BamHI/EcoRI) | AGCGGATCCGAATCATGGACAAAGAAACTAATAACC |
| GhGT23R(SalI/KpnI) | AGCGTCGACGGTACCACTTCTTCCTATCCTCAACG |
| GhGT23P1 | GCTAGTGGTTCTCTTCCTTTCT |
| GhGT23P2 | CTTCACACTGCATTGTACCG |
| U7F | AGAGGTCGAGTCTTCGGACA |
| U7R | GCTTGATCTTCTTG GGCTTG |
| ACT2F | GCACCCTGTTCTTCTTACCG |
| ACT2R | AACCCTCGTAGATTGGCACA |
| STZF | TCTCGCTCGCGACAACCGTC |
| STZR | GGCTTGCCTTGTGACCACCGA |
| COR6.6F | CCGCTGGCAAAGCTGAGGAGAA |
| COR6.6R | CCTCCCACTGCCGCATCCGATA |
| SAP18F | GCCAGTCTTCGCGAGCTAACA |
| SAP18R | AAGCCATCGTCTCCCCAACCT |
| COR47F | TCCCGGTACCAGTGTCGGAGA |
| COR47R | ACAACCAACGGCGTGGACGT |
| AP2F | AGGCGTGAGGCAAAGACCGT |
| AP2R | GCGGCTTCTTCTGCAGTGTCGA |
| DREB2BF | CAACAGCAACAGCTGCAACCGG |
| DREB2BR | GGTCTTGGCTCTGATGGGGACCA |
| DREB2AF | GACCGGTACCCGGGGAACAGT |
| DREB2AR | TCCCTCGAGCTGAAACGGAGGTA |
| RD22F | TACAAAATCGCGGCGGCTGGG |
| RD22R | AGCGGAACCGCGTAGACGGT |
| COR15AF | AGCTGAGAAAGCTGCGGCGT |
| COR15AR | TGGCATCCTTAGCCTCTCCTGCT |
| DREB2CF | ATTGGCTTATGACGAGGCGGCC |
| DREB2CR | AACCGAGCCTGACACAGTGGCA |
| COR47F | TCCCGGTACCAGTGTCGGAGA |
| COR47R | ACAACCAACGGCGTGGACGT |
